# Beyond acrodynia: infantile mercury vapor poisoning presenting with refractory hypoglycemia and persistent neurological damage

**DOI:** 10.1186/s12887-026-06950-z

**Published:** 2026-04-30

**Authors:** Shifan Wu, Qihan Yu, Jie Ying, Jiehong Xie, Kehan Wang, Guangfeng Liu, Hongbing Pang

**Affiliations:** 1 Ningbo Institute of Forensic Science, No. 11, Northwest Street, Ningbo, Zhejiang 315000 China; 2 School of Basic Medical Sciences, Hebei Medical University, 361 Zhongshan East Road, Shijiazhuang, Hebei 050017 China; 3 Xiangshan County Public Security Judicial Appraisal Center, Ningbo, Zhejiang 315700 China

**Keywords:** Infant, Mercury poisoning, Hypoglycemia, Persistent neurological damage

## Abstract

Infantile elemental mercury poisoning is clinically rare and poses a severe long-term health threat. This report describes a rare case of non-accidental infantile elemental mercury poisoning, in which the infant was continuously exposed to mercury vapor for 20 days starting from postnatal day 15. The infant initially presented with feeding difficulties and respiratory symptoms, which rapidly progressed to respiratory distress and seizure-like episodes at 5 months of age; laboratory tests revealed severe hypoglycemia and metabolic acidosis. Despite 5 days of intravenous glucose infusion and comprehensive support involving mechanical ventilation, sedation, and corticosteroids, hypoglycemia and epileptic symptoms persisted. The diagnosis of mercury poisoning was confirmed by a blood mercury test and governmental investigation, followed by two courses of dimercaptopropane sulfonate (DMPS) chelation therapy, after which hypoglycemia, respiratory symptoms, and epilepsy were effectively managed. The patient was discharged 25 days after admission. CT images revealed no abnormalities in the head at admission, but subsequent MRI at discharge and thereafter indicated persistent brain damage, including signal abnormalities and cerebral atrophy. This case suggests that severe hypoglycemia can be a critical component of infantile elemental mercury poisoning and contributes to persistent neurological injury. It also highlights that the efficacy of chelation therapy is severely limited when treatment is delayed after prolonged exposure. Current chelators such as DMPS have poor blood–brain barrier penetration and cannot effectively remove accumulated inorganic mercury from the CNS, underscoring the urgent need for novel CNS-penetrating therapeutic strategies in delayed presentations.

## Introduction

Infantile elemental mercury vapor poisoning is a clinically rare but critically severe condition, most often resulting from accidental exposure to damaged thermometers, fluorescent lamps, or other mercury-containing household items [1, 2]. Reports of infantile cases remain limited [3–5]. Mercury poisoning is generally classified into elemental, inorganic, and organic forms, which differ significantly in their metabolism, half-lives, and mechanisms of toxicity. Elemental mercury is highly volatile and lipophilic, facilitating its absorption into the bloodstream via the alveoli and its rapid penetration through the immature blood‒brain barrier. Once inside the central nervous system (CNS), it becomes effectively trapped. This prolonged accumulation induces persistent oxidative stress and neuronal apoptosis, ultimately leading to the profound and irreversible CNS damage that defines severe cases [2]. Compared with older children and adults, infants are at markedly higher risk under identical exposure conditions because of their greater minute ventilation per unit body weight, tendency to remain close to the floor where mercury vapor density is highest, and immature metabolic and glycogen reserves [2, 6]. This report describes an infant with elemental mercury vapor poisoning who presented with novel manifestations—severe refractory hypoglycemia and persistent brain injury—thereby expanding the known clinical spectrum of infantile elemental mercury exposure and highlighting the associated diagnostic and therapeutic challenges.

## Patient presentation

A 5-month-old male infant presented to the emergency department with respiratory distress and seizure-like episodes characterized by loss of consciousness, upward gaze deviation, trismus, limb rigidity, and incontinence. Laboratory investigations confirmed multisystem involvement, as evidenced by severe hypoglycemia (0.9 mmol/L, repeat capillary blood glucose: 2.9 mmol/L, normal 3.89–5.83 mmol/L), metabolic acidosis and myocardial injury (NT-proBNP 4242 pg/mL with normal 0–125.2 pg/mL, CK-MB 28.9 ng/mL with normal 0–5 ng/mL, and cTnI 0.168 µg/mL with normal < 0.028 µg/mL). Hepatic and renal function tests were within normal limits. The maximum glucose infusion rate (GIR) required on admission was 10 mg/kg/min.

**Fig. 1:**
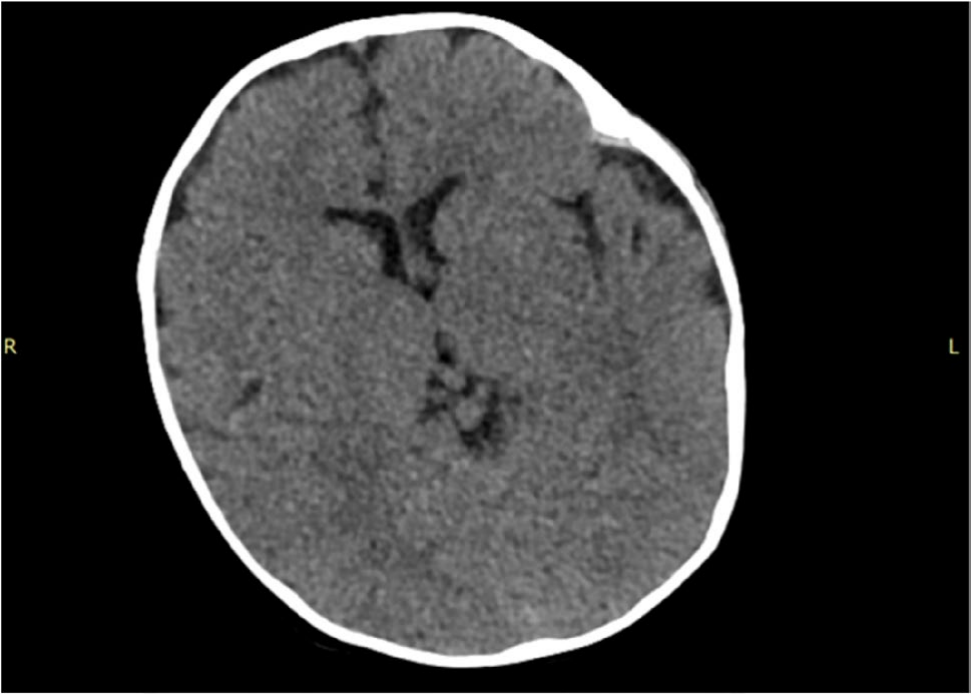
Head CT scan performed on admission showed no significant abnormalities

Over the first 5 days, investigations for common etiologies—including perinatal asphyxia, pulmonary and intracranial infections, and inherited metabolic disorders—yielded no explanatory findings. Notably, whole-exome sequencing (WES) revealed no pathogenic variants. Cerebrospinal fluid (CSF) examination showed clear fluid with normal cell counts (WBC 2/µL), normal protein and LDH levels, and no evidence of infection; however, the CSF glucose level was profoundly low (0.45 mmol/L), consistent with systemic hypoglycemia. Initial head computed tomography (CT) showed no significant abnormalities (Fig. 1). On day 5, electroencephalography revealed occasional spike waves but no definitive electrographic seizures. Despite intravenous glucose, corticosteroids, and respiratory support, the hypoglycemia and seizures remained refractory.

A further detailed history revealed a non-accidental exposure to elemental mercury vapors. Approximately 100 g of liquid mercury had been deliberately introduced into the infant’s 15 m² bedroom on two separate occasions during the neonatal period (postnatal days 15–35), contaminating the bedside table, bedding, and floor. Although a portion was removed during routine cleaning, the family remained unaware of the hazard until another relative identified the substance as mercury on day 35, at which point the infant was immediately relocated. The inhalation risk was significant as the infant spent most of the day indoors at approximately 19 °C. Clinically, a paroxysmal cough had emerged shortly after birth, which was initially misdiagnosed as “bronchitis” despite normal inflammatory markers. Just prior to admission at 5 months of age, an acute exacerbation occurred with sudden vomiting and poor feeding, despite previously normal growth parameters.

Blood mercury testing confirmed the diagnosis, showing markedly elevated levels in the infant (44.68 µg/L; normal < 14.9 µg/L) and the mother (15.57 µg/L). The source was confirmed through laboratory analysis of droplets from the residence and corroborated by government purchase records. Fasting insulin (26.4 µU/mL; normal 1.9–23 µU/mL) and C-peptide (6.6 ng/mL; normal 0.3–1.6 ng/mL) were markedly elevated prior to chelation (Table 1).

**Table 1:** Key biochemical parameters during hypoglycemia evaluation and response to chelation

Time point	Blood glucose (mmol/L)	GIR (mg/kg/min)	Insulin (µU/mL)	C-peptide (ng/mL)	Blood mercury (µg/L)	Comment
Admission (pre-chelation)	0.9 (repeat 2.9)	10	26.4	6.6	44.68	Peak values
Post-intensive phase	5.7	5	5.9	1.6	23.07	After first DMPS
Post-maintenance phase	5.7	0	8.3	0.6	7.26	Normalized
Reference range	4.1–5.9	-	1.9–23	0.3–1.6	<14.9	-

**Fig. 2:**
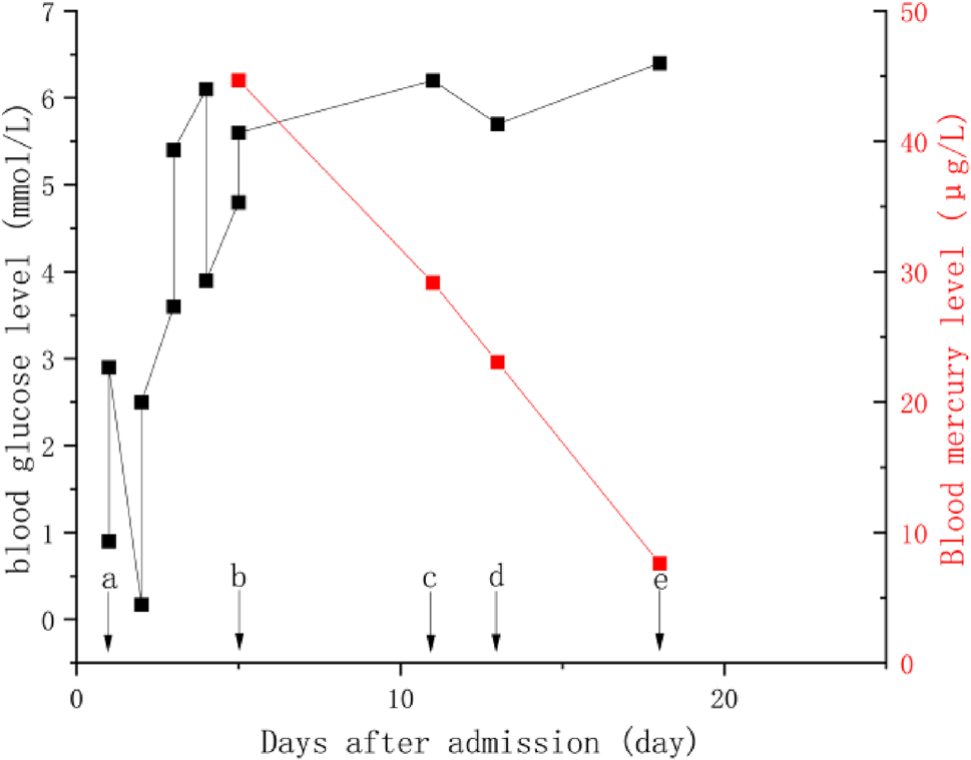
Timeline of blood mercury and glucose levels in the infant following admission. Blood mercury (red line, right axis) declined following the tapered DMPS regimen. Blood glucose (black line, left axis) stabilized following interventions. Dynamic changes of neurological symptoms and interventions: **a** Emergency admission. **b** Severe CNS symptoms (loss of consciousness, generalized tonic seizures, and incontinence); treated with steroids + IV glucose (10 mg/kg/min). **c** Partial recovery (normal muscle tone, occasional self-limiting focal seizures); treated with steroids + IV glucose (5 mg/kg/min). **d** The end of the first chelation. **e** Complete resolution (seizure-free, normal muscle tone); transitioned to steroids, formula, and oral glucose

**Fig. 3:**
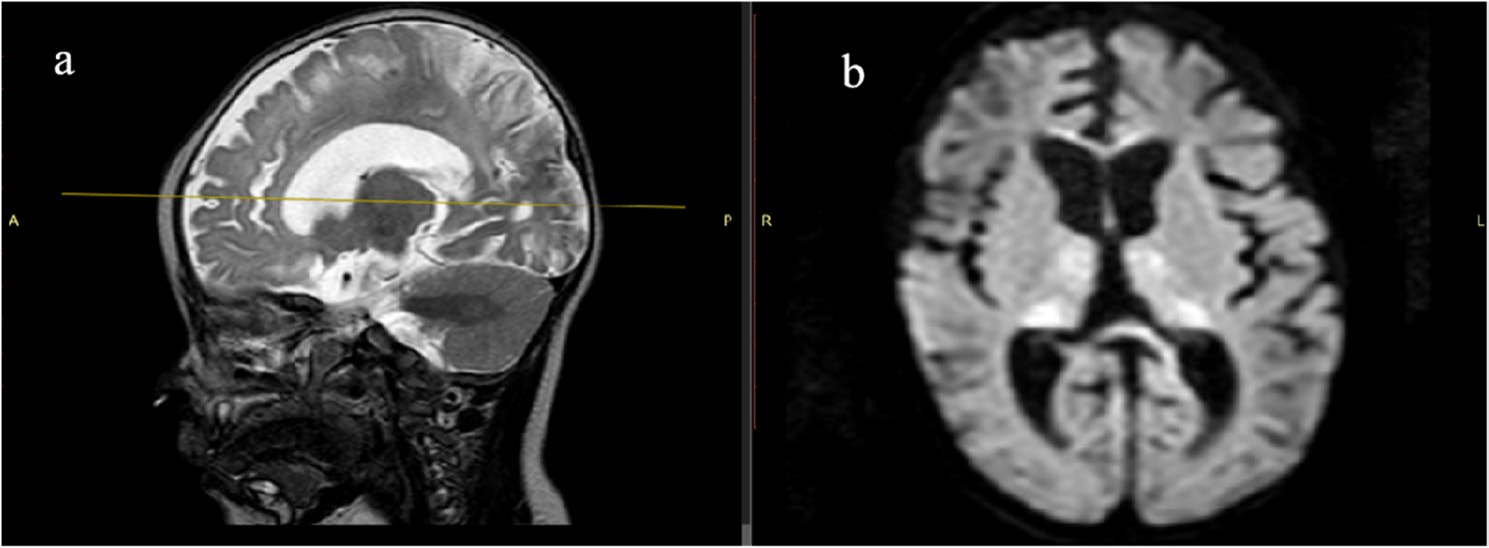
**a** Atrophy with multiple patchy T2 hyperintensities was observed in the cerebral hemisphere cortex. **b** DWI revealed abnormal signals involving the bilateral basal ganglia, thalamus, and corpus callosum

Following the diagnosis, DMPS chelation therapy was initiated (35 mg IV following a tapered regimen: q6h → q12h → q1d → q2d) according to Chinese national criteria (GBZ 89-2019). This prompted a favorable biochemical response, with blood mercury levels declining to 23.07 µg/L and 7.26 µg/L at subsequent intervals (Fig. 2). Urinary mercury concentration at the end of the first course was 138 µg/L, confirming effective mobilization. While acute symptoms and cardiac markers normalized, head MRI at discharge revealed persistent diffusion-weighted imaging (DWI) hyperintensities in the basal ganglia, thalamus, and corpus callosum, alongside bilateral cerebral atrophy and cortical encephalomalacia (Fig. 3), accompanied by sensorineural hearing loss.

**Fig. 4:**
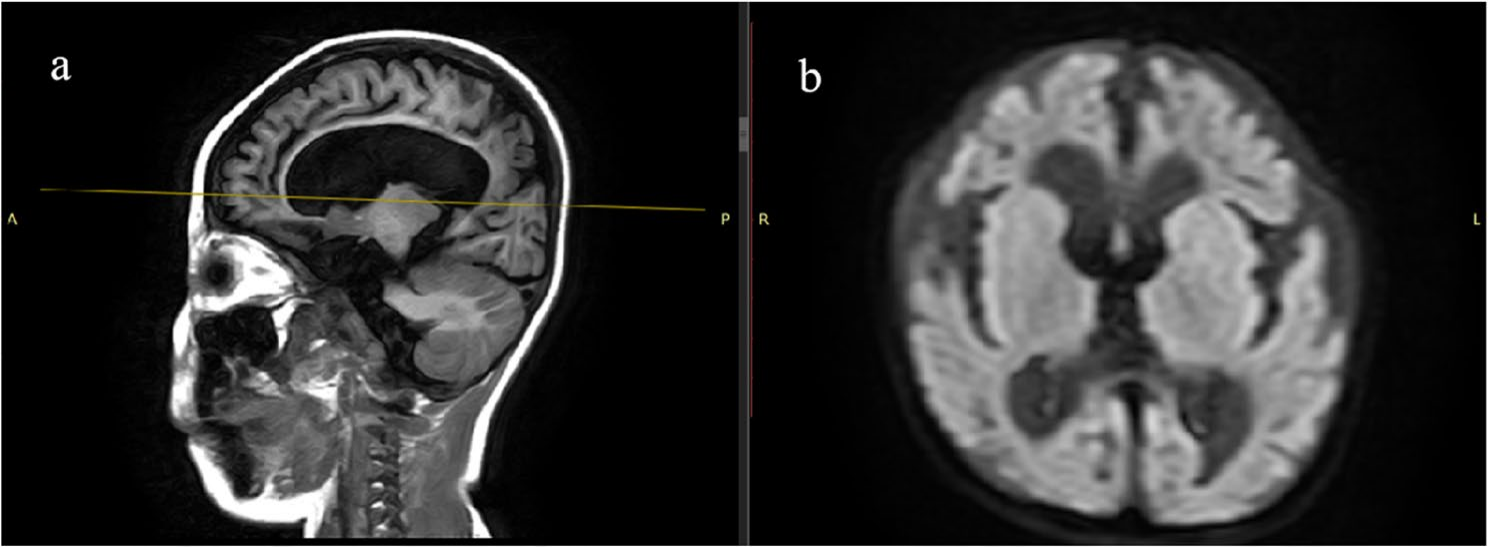
**a** The MRI reveals cerebral atrophy with reduced white matter volume, ventricular dilation, and thinning of the corpus callosum. **b** DWI showed resolution of the abnormal signals in the bilateral basal ganglia, thalamus, and corpus callosum

At the two-year follow-up, brain MRI confirmed irreversible structural damage, including frontotemporal subarachnoid space widening, sulcal deepening, ventricular enlargement, and reduced parenchymal volume (Fig. 4). Clinically, the patient exhibited significant global developmental delay and recurrent pneumonia. Chest imaging showed increased bronchovascular markings without radiopaque mercury deposits, and serial monitoring showed no rebound of blood mercury levels.

## Discussion

To our knowledge, this is the first reported case of infantile elemental mercury poisoning presenting with severe hypoglycemia and persistent brain injury. Unlike typical chronic mercury poisoning, our patient lacked common features such as acrodynia, rash, and hypertension; instead, the initial clinical picture suggested a pulmonary infection, which gradually progressed to respiratory distress and seizures over several months [2, 6].

As observed in other infantile cases, elemental mercury vapor exposure in this patient resulted in prominent respiratory and CNS involvement [3–5]. This multisystem toxicity is primarily driven by the unique pharmacokinetics of elemental mercury. Being highly lipophilic, it is rapidly absorbed through the alveoli and readily crosses the developing blood‒brain barrier. Once inside the CNS, it undergoes rapid oxidation into the highly reactive divalent ion (Hg²⁺). Because Hg²⁺ is poorly lipid-soluble, it becomes effectively trapped within the brain tissue. Here, it binds strongly to sulfhydryl (-SH) groups, disrupting functional proteins and inducing profound cellular damage [7].

The neurometabolic disturbances and irreversible supratentorial damage observed in this case highlight the distinctive pathophysiology of elemental mercury vapor exposure. Unlike classic organic mercury poisoning (e.g., methylmercury), which predominantly affects the cerebellum, visual cortex, and sensory pathways—leading to ataxia, visual field constriction, and sensory neuropathy [8], elemental mercury primarily causes diffuse cortical, basal ganglia, and thalamic injury with motor, cognitive, and developmental deficits. Furthermore, while isolated refractory infantile hypoglycemia typically results in brain injury predominantly affecting the parietal and occipital lobes, with bilateral occipital cortical injury being the most common pattern observed on MRI [9]. The MRI findings in this infant—DWI hyperintensities in the basal ganglia, thalamus, and corpus callosum combined with widespread cerebral atrophy—most closely resemble mercury-induced toxic encephalopathy. Nevertheless, the concurrent severe hypoglycemia and hypoxemia likely exerted a synergistic effect, exacerbating neuronal vulnerability through impaired energy metabolism and oxidative stress in regions already compromised by Hg²⁺ accumulation.

In addition to these neurological features, this patient developed refractory hypoglycemia. Interestingly, in contrast to the hyperglycemia commonly reported in mercury poisoning [10], this patient presented with severe hypoglycemia driven by transient hyperinsulinism. Prior to chelation, markedly elevated fasting insulin and C-peptide levels prompted an evaluation for congenital hyperinsulinism or insulinoma. However, a structured metabolic workup effectively excluded these common causes. The infant was normal at birth, and subsequent investigations—including β-hydroxybutyrate, ammonia, liver and renal function, abdominal imaging, and whole-exome sequencing—were unremarkable. We propose a multifactorial mechanism for this severe presentation. The infant’s physiologically limited hepatic glycogen stores, combined with acute vomiting and poor feeding in the days immediately preceding admission, created a state of metabolic vulnerability. The superimposed direct toxicity of mercury on pancreatic β-cells likely triggered inappropriate insulin secretion, precipitating refractory hypoglycemia even without evidence of chronic malnutrition [11]. This acquired, mercury-induced mechanism is robustly supported by the patient’s response to treatment. Following DMPS chelation, the hyperinsulinemia completely resolved. The clear temporal correlation between declining blood mercury levels and glycemic stabilization (Fig. 2), along with the normalization of insulin and C-peptide levels, strongly suggests mercury toxicity as the underlying etiology.

Mercury poisoning is a systemic disease that frequently induces multisystem dysfunction [12]. In this case, manifestations beyond hypoglycemia—including respiratory failure, seizures, metabolic acidosis, and myocardial injury—suggest that hypoglycemia is one component of widespread pathophysiological alterations. Animal studies have demonstrated that HgCl₂ can cause hypoglycemia and hyperinsulinemia via pancreatic β-apoptosis and aberrant insulin release [7]. Concurrently, the severe physiological stress induced by respiratory failure and systemic illness serves as a profound metabolic burden. In infants, who inherently possess limited hepatic glycogen stores, such hypermetabolic states rapidly deplete energy reserves and readily precipitate hypoglycemia [13]. Thus, the observed hypoglycemia likely resulted from combined direct pancreatic islets toxicity and secondary contributions from systemic toxicity.

Neuroimaging and long-term follow-up further underscored the severity of central nervous system involvement. Initial head CT was normal, whereas discharge MRI revealed diffusion-weighted imaging (DWI) hyperintensities in the basal ganglia, thalamus, and corpus callosum, accompanied by cerebral atrophy, gray‒white matter blurring, and multifocal cortical T2 hyperintensities. This is consistent with the subacute-to-chronic nature of elemental mercury vapor toxicity, in which structural changes may remain undetectable on CT even after prolonged exposure [5]. Atrophy and cortical softening indicate chronic neuronal loss and gliosis, whereas DWI changes reflect acute injury [14]. It is noteworthy that the admission blood mercury concentration of 44.68 µg/L, although only moderately elevated in absolute terms, is entirely consistent with these severe structural outcomes. Because elemental mercury rapidly crosses the underdeveloped blood‒brain barrier [15] and is oxidized to Hg²⁺, and becomes trapped within the CNS with an extended half-life ranging from years to decades [16, 17]—the blood level measured at 5 months of age likely represents the declining tail of a much higher early exposure burden. This severe CNS retention is further compounded by the limited permeability of DMPS across the blood‒brain barrier [18], leaving the accumulated cerebral mercury effectively beyond the reach of chelation therapy. Furthermore, the rapid CNS deterioration and profound supratentorial atrophy likely resulted from a synergistic “double-hit”: mercury-induced neuronal vulnerability compounded by acute energy failure from severe hypoglycemia and hypoxemia during a critical window of brain development [9, 19]. The 2-year follow-up confirmed severe neurological impairment with cerebral hypoplasia, growth and developmental delays, absence of voluntary movement and emotional expression, and profound language deficits consistent with mercury-induced CNS damage. In contrast to other poisoning patterns (Table 2), chronic elemental mercury exposure in infants typically leads to irreversible supratentorial atrophy rather than reversible or acute lethal manifestations.

**Table 2:** Comparative Analysis of Pediatric Mercury Poisoning Cases

Case/Age	Exposure Type/Duration	Key Symptoms	Imaging Findings	Treatment/Outcome	Persistent Damage	Reference
Our case	Elemental ; 20 days	Respiratory distress, hypoglycemia, acidosis, No fever, no erythematous rash	Cortical atrophy and T2-patchy hyperintensity, DWI-abnormal signals in bilateral basal ganglia, thalamus and corpus callosum on	Supportive care (IV/oral glucose, ventilation, hormones); DMPS, 2 courses; Hypoglycemia controlled, discharged day 25	Yes (Hemispheric atrophy, diffuse supratentorial changes)	N/A
3-Month-Old	Elemental ; Acute	Dyspnea, cough, pneumothorax, respiratory failure, no fever, no erythematous rash	Not detailed	CPAP ventilation, anti-infection treatment, nutrition support; DMPS; Full resolution	N/A	[3]
17-Day-Old	Inorganic ; Acute	Metallic urine, burns, cyanosis, pneumonia, shock; No fever, no erythematous rash	Not reported	Supportive care (ventilation, bicarbonate, intercostal drainage); D-penicillamine; Fatal	N/A	[4]
10-year-old	Elemental ; 20 days	Acrodynia, seizures, visual impairment, Hypertension	T2-hyperintensities in white matter/basal ganglia	D-penicillamine; Improvement	Partial	[5]
Pediatric cohort	Organic; Chronic	Cognitive/motor deficits, ataxia	Cortical atrophy, gliosis, cerebellar involvement	Variable; often irreversible	Yes	[9]
4-Year-Old	Inorganic; Chronic	Hypertension, restlessness, epilepsy, proteinuria	Subcortical white matter hyperintensities	DMPS; Full recovery	NO	[20]

Mercury can also induce myocardial damage [2, 6]. Although elevated cardiac enzymes were present, echocardiography and electrocardiography showed no structural or functional abnormalities—a discrepancy also noted in adult cases [21]. Increased enzyme levels in the absence of overt damage likely reflect subclinical myocardial stress or transient oxidative injury rather than permanent structural harm [22–24].

Collectively, the clinical course—from initial diagnostic ambiguity due to hypoglycemia to irreversible neurological sequelae despite chelation—expands the known spectrum of infantile mercury poisoning. It also underscores the current limitations of existing management strategies in preventing CNS outcomes. This gap between early recognition and effective neuroprotection remains a critical challenge and highlights the need for future research into novel therapies capable of targeting mercury accumulation within the central nervous system.

### Limitations

This single-case report has inherent limitations. The diagnosis of mercury-induced hypoglycemia, while clinically supported, remains inferential and precludes definitive causal conclusions. Furthermore, the severe neurological outcome is specific to this infant’s exposure context, limiting generalizable prognostic insights. These constraints highlight the challenge of translating these findings into universal clinical guidelines.

## Conclusion

Our observations raise the possibility that refractory hypoglycemia can be a potential, albeit atypical, manifestation of infantile mercury poisoning. Given this association, mercury exposure is worth considering in infants with unexplained multisystem involvement. The failure of chelation to prevent CNS damage highlights the limited efficacy of current therapies against brain mercury burden, underscoring an urgent need for novel strategies capable of crossing the blood-brain barrier.

## Data Availability

The genetic data are clinical diagnostic records, not primary research data subject to mandatory deposition. The negative finding is reported in the text. Relevant anonymized information is available from the corresponding author upon request.

## Abbreviations

CK-MB: Creatine Kinase-MB
cTn I: Troponin I, CNS: central nervous system
Hg²⁺: mercury ion
IV: Intravenous injection
CPAP: Continuos Positive Airway Pressure
DMPS: Dimercaptopropane sulfonate
DWI: Diffusion-weighted imaging
GIR: Glucose infusion rate
Hg²⁺: Divalent mercury ion
IRB: Institutional Review Board
N/A: Not Applicable
NT-proBNP: N-terminal pro-B-type natriuretic peptide
CSF: Cerebrospinal fluid
